# Optofluidic Device Based Microflow Cytometers for Particle/Cell Detection: A Review

**DOI:** 10.3390/mi7040070

**Published:** 2016-04-15

**Authors:** Yushan Zhang, Benjamin R. Watts, Tianyi Guo, Zhiyi Zhang, Changqing Xu, Qiyin Fang

**Affiliations:** 1 School of Biomedical Engineering, McMaster University, 1280 Main Street West, Hamilton, ON L8S 4L8, Canada; zhang749@mcmaster.ca (Y.Z.); guot2@mcmaster.ca (T.G.); 2 ArtIC Photonics, 260 Terence Matthews Cres, Ottawa, ON K2M 2C7, Canada; benjamin.r.watts@gmail.com; 3 Information and Communication Technologies, National Research Council of Canada, 1200 Montreal Road, Ottawa, ON K1A 0R6, Canada; zhiyi.zhang@nrc-cnrc.gc.ca; 4 Department of Engineering Physics, McMaster University, 1280 Main Street West, Hamilton, ON L8S 4L8, Canada; qiyin.fang@mcmaster.ca

**Keywords:** optofluidic device, microfluidics, microflow cytometer, microfabrication

## Abstract

Optofluidic devices combining micro-optical and microfluidic components bring a host of new advantages to conventional microfluidic devices. Aspects, such as optical beam shaping, can be integrated on-chip and provide high-sensitivity and built-in optical alignment. Optofluidic microflow cytometers have been demonstrated in applications, such as point-of-care diagnostics, cellular immunophenotyping, rare cell analysis, genomics and analytical chemistry. Flow control, light guiding and collecting, data collection and data analysis are the four main techniques attributed to the performance of the optofluidic microflow cytometer. Each of the four areas is discussed in detail to show the basic principles and recent developments. 3D microfabrication techniques are discussed in their use to make these novel microfluidic devices, and the integration of the whole system takes advantage of the miniaturization of each sub-system. The combination of these different techniques is a spur to the development of microflow cytometers, and results show the performance of many types of microflow cytometers developed recently.

## 1. Introduction

Since the original attempt in 1934 when researchers first successfully counted particles and cells in a small tube [1], flow cytometry has developed into a powerful technique for cell analysis, sorting and counting. Recently, flow cytometry has been applied in many fields, such as point-of-care (POC) diagnostics, cellular immunophenotyping, rare cell analysis and genomics [2]. The commercialization of conventional flow cytometers has been very successful: the market of modern microflow cytometers is expected to reach $3.6–5.7 billion by 2018 at a compound annual growth rate of 18%–29% [3]. Compared to a bulky conventional flow cytometer, microchip-based flow cytometers (referred to as microflow cytometers in this paper) are simple to use, time efficient, consume low amounts of expensive reagents and have overall lower associated costs (capital, operation, training, *etc*). With the rapidly developing demands of POC applications, the growing demands of *in situ* and *in vitro* diagnostics in the biomedical field and the need to improve rapid analysis and synthesis in the chemical field, microflow cytometers are poised to facilitate great advancement in these and other fields and allow applications that will change many aspects of everyday life in the near future.

The term “optofluidics” was first mentioned in 2003 and was coined to reference new devices that integrated the fields of optics and microfluidics [4]. Microfluidics is the technology that manipulates fluids on the nL–fL scale on a microchip platform, whereas optofluidics manipulates both fluids and optics simultaneously in a seamlessly integrated platform. A microchip-based device that is based on the technology of microfluidics is called a microfluidic device, while an optofluidic device is a device based on optofluidics, requiring both fluidic and optical capabilities. A microflow cytometer is a highly integrated system that utilizes a microchip-based device for the fluidic handling and manipulation in a flow cytometry application. An optofluidic microflow cytometer utilizes an optofluidic device to apply flow cytometry using a single device to integrate both the fluidic and optical sub-systems onto a single device. Classification of the terminology and the function of the device is shown in Table 1.

In an optofluidic microflow cytometer, the optical components are integrated into a microfluidic system and *vice versa* [5]. Integration allows the benefits of including new optical features on the device, such as built-in optical alignment, beam shaping, high optical sensitivity and tenability-each seamlessly integrated in one platform with fluidics. Previous review papers have already summarized the fundamentals and applications of optofluidic technology [4,5,6]. In this paper, we will discuss the basic principles and components of an optofluidic device-based microflow cytometer in detail, as well as review the performance of a few microflow cytometers developed recently and compare the performance of the devices.

### 1.1. The Principles of Flow Cytometry

Flow cytometry is a powerful analysis technology for the characterization of cells or particles. Multiple parameters, *i.e.*, size, shape, cell granularity and cell viability, can be detected simultaneously at rates of up to 50,000 particles per second [1]. The original intention of flow cytometry was to measure particles or cells one-by-one as they passed through a laser beam in a single file stream flowing through a glass tube [7]. Scattered light at both small and large angles, as well as fluorescence light (FL) emitted from fluorescent labels are detected and analyzed for every single cell or particle. Light scattered at a small angle from the input beam axis is referred to as forward scattered light (FSC), whereas large angles of scattered light are called side scattered light (SSC). The intensity of FSC is generally determined by the size of the cell, while the granularity of the particle or cell determines the intensity of SSC. Through the analysis of the two data parameters, the cells or particles can be identified, counted and sorted downstream. The performance of a flow cytometer is dependent on one of the four main techniques integral to flow cytometry: particle focusing, beam shaping, signal detection and data analysis.

In flow cytometry, particle focusing is applied to ensure the cells of interest pass through the optical interrogation point one by one, reducing the possibility of a double detection. The interrogation region is the intersection between the excitation light beam and the solid angle accepting scattered light or fluorescence from the detection optics. The sample fluid containing cells or particles is surrounded by a sheath fluid which confines the particles to a narrow stream in the center of the channel, roughly one cell or particle in diameter.

Conventional flow cytometers focus and shape the excitation beam by using a free-space lens system. Ideally, the beam would be aligned with focused sample stream, and the beam width would be no less than the width of sample stream to ensure the entire particle or cell can be illuminated. In addition, the portion of the light beam outside the sample stream is minimized to keep the background illumination, thus the noise on the detection channels, as low as possible.

A pulse is produced when a particle or cell passes through the laser beam. The pulse shape and amplitude relates to the interaction between the incident beam and the particle or cell, including the incident light intensity, particle size, geometry and granularity, as well as the fluorescence efficiency. The pulse duration depends on the beam width and linear velocity of the particle along the channel. Therefore, light beam intensity with a super-Gaussian distribution along the flow direction is preferred to generate pulses close to a square waveform.

When particles pass through the laser beam, SSC, FSC and fluorescence light signals are detected by a number of detectors. A modern flow cytometer can detect as many as 17 independent channels featuring a combination of several FL wavelengths, FSC and several different angles of SSC simultaneously by using a series of dichroic mirrors [1]. Thus, multi-parameters can be monitored by analyzing those light signals. In some circumstances, in conjunction with or in place of an optical interrogation method, impedance-based cell or a particle sorting, counting and differentiating method can also be applied in flow cytometry [8,9]. Cells or particles pass through a small area enclosed by two electrodes, where electrophysiological impedance variation can be detected for every single cell or particle.

### 1.2. Microflow Cytometer

Although conventional flow cytometers have gained profound success in cell sorting and analysis, they are bulky, demand large amounts of expensive reagents with complicated processing steps, are complicated in manipulation and require high maintenance costs. Typically, a flow cytometer will be located in a single facility where many hundreds of users will need access to it. These limitations restrict their uses in POC diagnostics, *in situ* pathogen monitoring and other application where portability, handling small volume samples, low operation costs and ease of operation are essential.

Owing to the recent development of lab-on-chip (LOC) technology, microfabrication and micromachining techniques, the miniaturization of a flow cytometer can be achieved. The microfabrication of a flow cytometer with 3D microstructures can be accomplished by 3D microfabrication techniques utilizing UV lithography [10]. Microfluidic mixing [11], microfluidic cell sorting and the miniaturization of pumps and valves [12] provide a basic foundation for miniaturizing the fluidic handling to develop a microchip-based flow cytometer. Researchers have been able to take advantage of these advanced microfabrication technologies to miniaturize a flow cytometer to a microscale or even a nanoscale platform. Controlling fluids in a microchannel allows microchip-based flow cytometer to be applied in POC diagnostics and lab-on-a-chip devices offers unique advantages [13], such as reducing the volumes of reagents, shortening the turnaround time between inspection and results and lowering the associated costs [14].

To date, the throughput of microflow cytometers can reach up to 50,000 cells/s [1], allowing microflow cytometers to have many applications: Titmarsh *et al.* [15] discussed how microfluidic technology spurred on the development of stem cell-derived therapies. Hashemi *et al.* [16] successfully distinguished different populations of phytoplankton with high sensitivity by measuring light scatter and fluorescence properties by a microflow cytometer. More demonstrations on diagnostic and point-of-care applications have been addressed in recent review papers [14,17].

A novel optofluidic device-based microflow cytometer emerged recently. Optical components and novel liquid lenses are used in microfluidic devices. Liquid-core/liquid-cladding waveguides and liquid core/air-cladding lens systems with larger refractive index contrast lead to less propagation losses and resulted in better optical confinement [18]. Additionally, an integrated on-chip lens system or grooved on-chip fibers further reduce the size of the microflow cytometer. Built-in waveguides also are free of optical alignment, making operation much easier. Recently, Liang *et al.* took advantage of evanescent waves present at the liquid-liquid interface of immiscible flows to count the nanoparticles on an optofluidic microchip [19].

## 2. Major Components of an Optofluidic Microflow Cytometer

Typically, the creation of an optofluidic device-based microflow cytometer includes four principle design areas: (1) the flow control; (2) the optical design; (3) the microfabrication of functional layers; (4) the integration of the entire system. The flow control includes how to bring flow into a device and to ensure cells or particles are being focused in the interrogation region, which is usually achieved by 2D or 3D hydrodynamic focusing methods. The optical system provides light for interaction and collects light signals for analysis. The microfabrication of fluid control and optical components provide a microscale or nanoscale platform for cell analysis. System integration includes the miniaturization of the device and provides user-friendly control environment and easy-to-use data analysis software.

**Figure 1:**
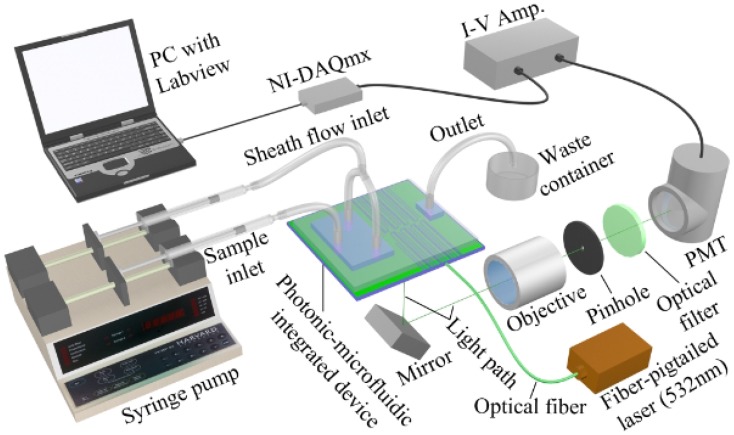
System setup of an optofluidic device (also referred to as photonic-microfluidic integrated device)-based microflow cytometer. Laser light is focused through on-chip lenses, and side scattered light (SSC) and fluorescence light (FL) signals are detected via free-space lens system. Light signals are amplified by a photomultiplier tube (PMT), then data are analyzed by a data acquisition card (DAQ). Reproduced from [20] with kind permission from Wiley.

Figure 1 shows a system setup of a typical optofluidic microflow cytometer [20]. Cells are delivered to the interrogation region in a sample fluid surrounded by two sheath fluids. Cells traverse the light in the interrogation region that has been focused by the on-chip lens system and produces its characteristic optical signature containing SSC, FSC and FL signals. In this iteration of the device, the collection arm is not integrated on the device like the excitation optics, and thus, the signals are collected via a free space objective and directed to a spectral and spatial filter where they are finally detected and amplified by a photomultiplier tube (PMT). In this device, the bulky and expensive free space optical lens system for excitation in a conventional flow cytometer was replaced by cost-effective, space-saving and free optical alignment on-chip lens system.

The challenge and difficulty of miniaturizing the flow cytometer is how to make the performance of a microflow cytometer comparable to the conventional benchtop flow cytometer. In this chapter, optofluidic microflow cytometers with different features classified in Table 2 will be discussed. All of the aspects in Table 2 contribute to the performance of an optofluidic microflow cytometer and will be discussed in detail in later sections.

### 2.1. Flow Control

In microfluidics, the sheath fluids and sample fluid can be considered as Newtonian fluids, which are continuous, laminar and incompressible. In microflow cytometers, passive flow driven by capillary force or gravity or active pumps driven by an external power source are used to provide continuous flow through the devices [21,22]. Since the cross-section of the channel on the scale of a few 10s of micrometers and the sidewalls are smooth, the flow in the microchannels can be classified as a Stokes flow with a Reynolds number less than one, meaning that the flow in the channel is in the laminar regime. Small dimensions of the microchannel may raise the risk of clogging by big particles or cells, clumps of cells or even extraneous debris. Details about basic concepts, fabrication strategies and advanced applications of hydrodynamic focusing in microflow cytometers can be found in a review by Ainla *et al.* [23].

#### 2.1.1. 2D Hydrodynamic Flow Focusing

**Figure 2:**
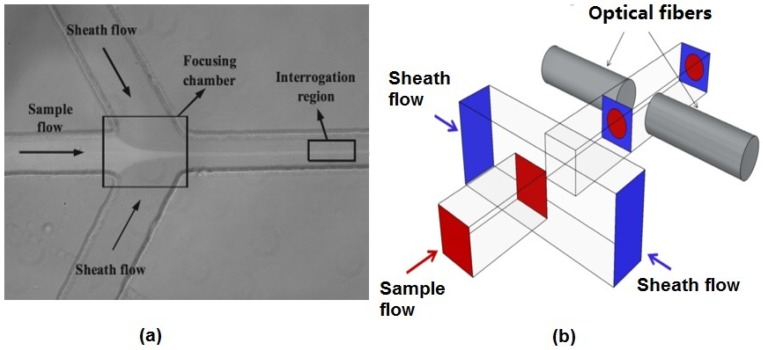
Reported hydrodynamic focusing methods in a microflow cytometer. (**a**) A typical structure of 2D hydrodynamic focusing. Reproduced from [24]. (**b**) A straight forward 3D hydrodynamic focusing structure. Reprinted from [31] with kind permission from OSA Publishing.

The hydrodynamic focusing technique used in both benchtop and microflow cytometers is one of the most successful and ubiquitous flow focusing techniques. The sample fluid is sandwiched between a sheath fluid in both sides, and since the Reynolds number is low and the fluids are in the laminar regime, there will be no turbulent mixing of the fluids. Figure 2a shows a typical structure used to achieve 2D hydrodynamic focusing in a microflow cytometer [24]. Similar structures that narrow the sample fluid between two sheath fluids have been widely used in microflow cytometry [16,25,26,27,28,29]. The width of the focused sample stream is related to the ratio of sample to sheath flow rate and effectively allows the user to tailor the sample stream width to the application’s requirements [30]. The sample stream’s width must be large enough to accommodate the largest particles in the sample population, yet not too large as to allow particles to flow side-by-side in the sample stream. It must be noted that the vertical channel height defines the height of sample fluid in 2D hydrodynamic scheme, and thus, careful consideration must be taken as to the channel height and the characteristic size of the cells or particles under inspection.

#### 2.1.2. 3D Hydrodynamic Flow Focusing

3D hydrodynamic focusing confines the sample fluid to the center of the microchannel in both vertical and horizontal directions, an improvement on the inability of 2D hydrodynamic focusing to focus fluid in the channel’s vertical dimension. The straightforward way to achieve 3D hydrodynamic focusing is to use deeper orthogonal sheath fluids, as shown in Figure 2b [31]. Two lateral fluids get in above and below the sample fluid in addition to lateral directions and push the sample fluid from both vertical and horizontal directions in a microchannel with a larger dimension than that of the sample channel. Since deeper channels are difficult to fabricate, requiring three fabrication and two alignment steps, 2D hydrodynamic focusing and its one fabrication step is preferred. However, a strategy of using 2D hydrodynamic focusing twice has been applied to achieve 3D hydrodynamic focusing. By using a planar structure, two sheath fluids A and B can be used to focus the sample fluid vertically, and the sample fluid was focused horizontally by another sheath fluid, C [32]. Experimental results and numerical simulation results show that the sample stream is focused to a small region in the center of the microchannel.

With 3D microfabrication technology, more complex structures are fabricated to focus the particles in two dimensions, such as oblique cylinders or grooves [10]. Sundararajan *et al.* used the “membrane sandwich” method, which contained two sheath fluids from lateral directions and another two on top and at the bottom stacked on the inlet point to create a 3D hydrodynamic focusing microchip [33]. Hairer *et al*. focused the sample fluid by using three sheath fluids in a non-coaxial sheath flow device [34]. V-shaped or chevron-shaped grooves were fabricated in microchannels to focus the sample fluid in both lateral and vertical directions [35,36]. Similar V-shaped slants were also applied in 3D mixing [37]. More recently, Nawaz *et al.* achieved 3D hydrodynamic focusing by using microfluidic drifting with different curvature angles [38].

#### 2.1.3. Other Methods

**Figure 3:**
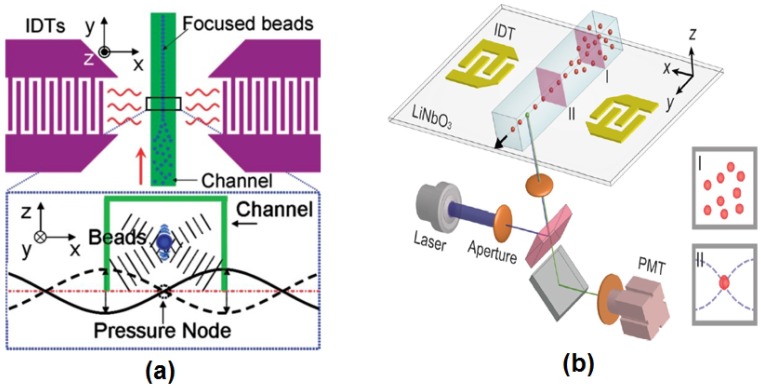
(**a**) A schematic diagram of standing surface acoustic waves (SSAW) focusing. The pressure node located at the center of the microchannel was generated by the SSAW field created by two parallel interdigital transducers (IDTs). When particles or cells enter the SSAW field, the acoustic radiation force (vertically and horizontally) moves the particles to the pressure node. Reprinted from [39] with kind permission from Royal Society of Chemistry. (**b**) A schematic diagram of a SSAW-based microflow cytometer. Reprinted from [41] with kind permission from Royal Society of Chemistry.

Besides hydrodynamic focusing, acoustics [39,40,41,42,43], dielectrophoresis (DEP) force [44,45,46,47], electrokinetics and magnetophoresis (MAP) [48,49,50] can be used alternatively to focus particles and cells in a microflow cytometer. Acoustic-based focusing methods do not need sheath fluids for 3D focusing. The standing surface acoustic waves (SSAW) field generated by two parallel interdigital transducers (IDTs) applies lateral and vertical acoustic radiation force to the particles or cells, as shown in Figure 3a. Particles or cells are focused in the center of the microchannels where the pressure node is located. Recently, Chen *et al.* created an SSAW-based 3D focusing microflow cytometer [41], as shown in Figure 3b.

Dielectrophoresis (DEP) is another approach to achieve 3D focusing in microchannels without sheath fluids. In DEP, a non-uniform oscillating electric field creates a dipole on the cells or particles that will experience a negative or positive force depending on the dipole’s phase with the applied AC field and the strength of the electric field at each end of the dipole. By changing the frequency of the field, it is possible to tune the strength of the force on the particles or even to switch the direction of the DEP force. The DEP force can adjust particle’s or cell’s equilibrium position-normally at the center of the channel in the vertical position by utilizing a pair of parallel microelectrodes on the top and bottom surface of the microchannel. Usually, particles or cells experience a negative force as a positive DEP force pulls the particles or cells towards the surface of the electrode where the greatest field gradient occurs that could destroy the cells [51]. The DEP force depends on the size and electrical properties of the particles or cells, the electrical properties of the sample fluids and the electric field. Large particles or cell move slower than smaller particles or cells, and the focusing pattern of each cell or particle is different. Many microchip-based flow cytometers now use more than one technique to confine sample flow. Lin *et al*. [52] combined the 2D hydrodynamic focusing and DEP method to obtain 3D focusing: two electrodes exerted a DEP force from the vertical direction on the particles and cells, which had already been focused laterally hydrodynamically by two sheath fluids. More recently, Zhang *et al.* presented a novel DEP-inertial microflow cytometer, which combined the DEP force and inertial force to achieve vertical-focusing [46]. The MAP theory is similar to DEP force, except that the electric field is replaced by the magnetic field. In addition, particles or cells need to be attached to a magnetic bead so that they can move to the interrogation point exactly.

### 2.2. Light Guide and Collection

Particles or cells focused in the center of the microfluidic channel are interrogated by a light beam. In a conventional flow cytometer, a light beam is guided to the capillary tube for excitation, while the various light signals are collected by an objective lens and subsequently split and detected in free-space by bulky optical lenses, dichroic mirrors and other components. A first step towards an optofluidic microflow cytometer involved moving the excitation optics to the chip. Integrating lenses onto the chip eliminates the need for free-space optical alignment while reducing the size of the microflow cytometer device and making the device more portable and durable. Free-space light collection was very similar to the conventional flow cytometer, as shown in Figure 1 [20]. This section will focus on the simulation and design of integrated on-chip optical systems for optical excitation in an optofluidic microchip-based flow cytometer.

#### 2.2.1. Excitation Sources and Optical Fibers

As shown in Figure 1, a laser beam is coupled into a fiber, and the fiber subsequently couples light to an integrated waveguides on the chip to precisely deliver the light to the channel to excite the FL of the particles or cells and generate the scatter signals [20]. In some research papers, light-emitting diodes (LEDs) and laser diodes are used as a source, and either could be used; however, light from an LED is noncoherent light with a wide bandwidth, but has a low cost, while the light from a laser diode is coherent light with a narrow bandwidth and has a higher cost. The correct source can be selected based on the application.

**Figure 4:**
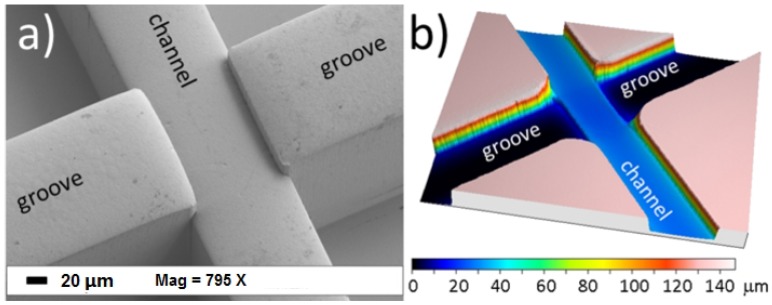
(**a**) SEM microgram of microgrooves for optical fibers. (**b**) confocal microscope profilometry of the microchip that shows the depths of microchannel and microgrooves. Reproduced from [56] from *Micromachines* published by MDPI.

Optical fibers (both single-mode fibers and multi-mode fibers) can be coupled with lasers to provide decent beam shaping at the interrogation region; however, the beam diverges as it leaves the guiding medium. Optofluidic devices coupled with single-mode fibers have been well-reviewed by Blue *et al.* [53]. A device with an integrated on-chip lens system couples light from an optical fiber to an on-chip waveguide to deliver the light to the lens system and focuses and shapes the light in the interrogation region [54,55]. Instead of integrated waveguides on the chip, microgrooves fabricated in the functional layer can help embed optical fibers into the microchip eliminating the need to align fibers to the chip. These inserted fibers can be used for the collection of light signals, as well. Inserted fibers ensure a complete optically-guided approach, from source to detector [2,26,56]. Matteucci *et al.* [56] fabricated grooves for the insertion of optical fibers to achieve precise alignment of optical power (as shown in Figure 4a).

#### 2.2.2. Waveguides

Optofluidic waveguides, such as solid-core/liquid-cladding waveguides (SCLC), liquid-core waveguides (LCW) [57,58] and hybrid core waveguides (HCW) [59,60], have been reported. Guiding of the light is ensured if the refractive index of the core material is higher than that of the cladding. In an optofluidic microflow cytometer, deionized (DI) water, water-based liquids and organic-based liquids are typically used for cladding materials, while glasses, polymers and semiconductors are typical materials used for the core. Choi *et al.* [61] used DI water as a core fluid and 2,2,2-trifluoroethanol as cladding fluid to form a waveguide. Liquid-core/air-cladding (LA) waveguides have been integrated into an optofluidic device by Lim *et al.* [62]. Shi *et al.* [60] demonstrated a hybrid waveguide consisting of a liquid-liquid waveguide and a liquid-solid waveguide to achieve real-time self-imaging in a microchannel. Compared to 2D liquid-liquid waveguide, the 3D liquid-liquid waveguide is surrounded by cladding fluid in both directions, and the confinement of light is better [63]. Yang *et al.* demonstrated bending and manipulating light via optofluidic waveguides with their unique optical properties [64].

**Figure 5:**
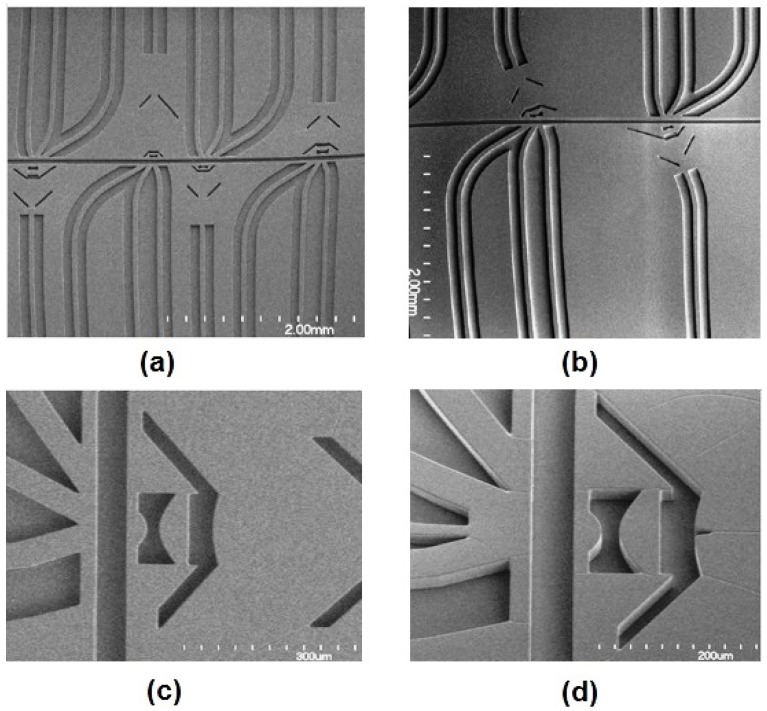
(**a**) SEM images of four microflow cytometers with different optical systems. Long straight waveguides direct lights to the lens system; waveguides at different angles are used to collect signals. (**b**) Images of angled input waveguides and the lens system to reduce background noise for SSC and FSC detection. (**c**) SEM images of on-chip air lens system without notches. (**d**) SEM images of on-chip air lens system with notches. Reproduced from Watts [65] with permission.

Optically transparent photoresists, such as SU-8, are widely used to fabricate on-chip waveguides integrated simultaneously with the microfluidic channel during the fabrication process [54]. SU-8 can function as the core while voids provide air to function as cladding material. As the refractive index of SU-8 (about 1.59) is higher than that of the air, strong optical confinement is observed, lowering the background noise [65]. Figure 5a shows a typical air cladding SU-8 core waveguide fabricated by Watts *et al.* [65]. Light is guided through the long straight waveguide to the lens system, shaping in the microchannel and then collected by multiple waveguides at angles at 5°, 30° and 75° to the input laser beam axis. FSC, FL and SSC are collected from each of the angled waveguides (respectively), where the angled on-chip waveguides help to avoid noise due to a couple of stray light signals from the input laser diode. To further improve the signal to noise ratio (SNR), an angled input waveguide along with an angled lens system were used to reduce the noise by allowing a full 90° angle between the input and SSC, as shown in Figure 5b. A low background noise is created for SSC, which has the same wavelength as that of the excitation beam. Waveguides made by optically transparent polymers, such as poly(dimethylsiloxane) (PDMS), can also be integrated onto the device. In addition to polymers and photoresists, researchers have shown that other materials can also function as waveguides. For example, Emile *et al.* [66] used 1D soap films as waveguides to guide light coming from a laser diode.

#### 2.2.3. On-Chip Lens System

**Figure 6:**
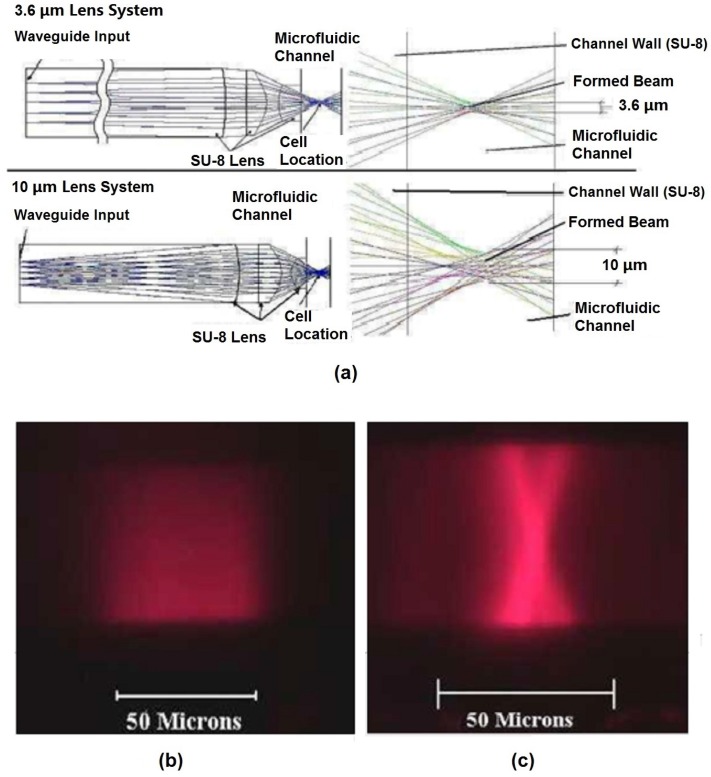
Beam shaping simulation results and fluorescent images of formed beam shape. (**a**) ZEMAX simulation results of 3.6-µm and 10-µm on-chip beam shaping lens systems. (**b**) Fluorescent image of the input excitation beam input directly from a waveguide without any lens. (**c**) Fluorescent image of formed beam shape after passing through a 10-µm beam shaping lens system. Reprinted from [67] with permission from OSA Publishing.

Due to the large numerical aperture of the on-chip waveguide inherit in the materials’ large index contrast, the beam coming out of the waveguide will diverge and expand as it traverses the distance from the waveguide facet to the interrogation region. In other words, the light that propagates to the interrogation region will have a large spot size and poor uniformity. For an optofluidic microflow cytometer, on-chip lens systems can be integrated in the microchip to replace bulky and expensive optical components used in free-space solutions. Beam shaping is used to focus the laser beam via a 2D lens system embedded on the microchip between the waveguide and the microchannel, increasing the uniformity of the light for interrogation (Figure 5). Watts *et al.* [54,55,65,67,68,69,70] reshaped the beam from the excitation laser to an optimized geometry in the interrogation region to enhance detection. Beam shaping process specifically reshaped the input laser spot geometry to a designed spot geometry where the center portion of the laser beam was altered to obtain a much smaller and uniform beam. For example, by adjusting design parameters of each surface in the lens system, a beam size of 1.5 µm and 3.6 µm, defined by the full width at half-maximum (FWHM), was formed at the focusing point in the microchannel [65]. The initial beam waist was around 50 µm, which was almost 33 times larger than the reshaped beam waist, and the added benefit of a significant corresponding increase in the beam intensity is also achieved. Simulations using commercial ZEMAX software (2005) shown in Figure 6a demonstrate the process of the shaping the beam from the input to output. Light emitted from the laser passes through the SU-8 waveguide, propagates along the SU-8/air lenses, and forms beam waists of 3.6 µm and 10 µm in the two examples shown. Fluorescent images of the beam with and without shaping (Figure 6b,c, respectively) show that the waveguide without integrated lenses have no control over the beam geometry. It is worth noting that the measured coefficient of variation (CV) of fluorescent beads was strongly dependent on the beam geometry and bead sizes: 2.5-µm fluorescent beads had the best CV of 8.5% for a 3.6-µm beam waist [65].

**Figure 7:**
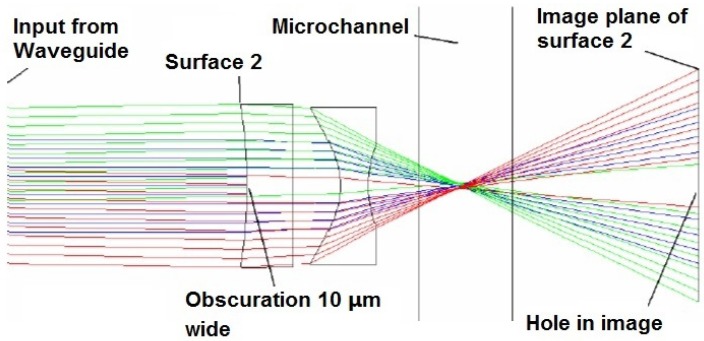
ZEMAX simulations for a 3-µm lens system that inserts a notch for forward scattered light detection. Note how the notch is re-imaged on the waveguide behind the channel. Reprinted from [65] with permission.

In conventional flow cytometry, a thin obscuration bar located before the detector, onto which the input beam is focused, is used to block the laser beam from reaching the detector directly. This technique is done because the laser beam propagates along the same axis that the FSC and obscures the FSC signal. In an optofluidic device, a notch was applied in the first surface of the lens system, functioning as an obscuration bar, as seen in Figure 7 [65]. The notched lens system forms a dark spot on the facet of a collection waveguide without influence on the beam geometry in the interrogation region. This notched design enhanced the SNR and improved the reliability of on-chip detection for FSC: results show that a false positive rate as low as 0.4% can be achieved [70].

In some optofluidic microflow cytometers, researchers combined the advantages of both liquid and light. Tang *et al.* [18] created a reconfigurable liquid-core/liquid-cladding lens ($L^{2}$ lens) formed by three laminar flows. Very similar to the air lens system, two streams of a lower refractive index function as the cladding, and a stream of a higher refractive index functions as the core. The focal length of the lens can be changed in real time by changing the relative flow rates of the three streams without mechanical moving parts. At the same time, the liquid lenses provide an optically-smooth interface for light manipulation. Those novel optofluidic components provide new opportunities for on-chip flow cytometers and cross the boundary of multiple disciplines.

**Figure 8:**
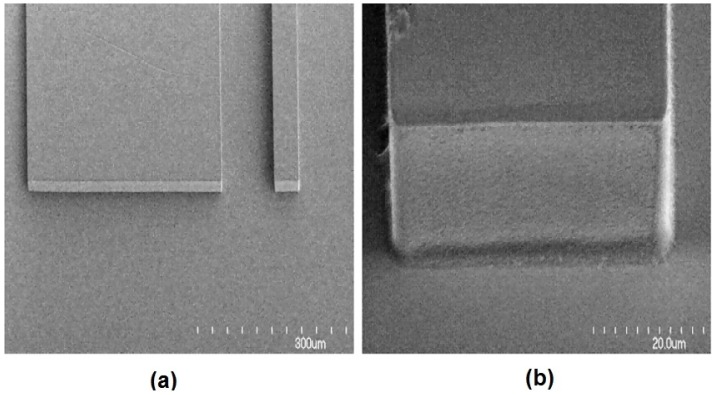
(**a**) SEM image of a waveguide facet. (**b**) A close-up SEM image of a waveguide facet showing the smooth optical coupling face. Reprinted from [65] with permission.

To minimize losses in the system, it is important to match the size of the on-chip waveguide and coupling fiber. The roughness of the waveguide, lens surfaces and channel wall can also cause propagation losses in the device. The SEM image in Figure 8 shows that the sidewall and facets are very smooth. The excellent quality of the waveguide facet, channel wall and lens surfaces allow low amounts of escaping light due to scattering from imperfection in the photolithographically-formed side walls. To further improve the sensitivity of the device, higher input laser power can be provided to enhance the SSC or FL signal of the particles or cells. The effect of roughness of the on-chip lens system can be ignored due to the high signal-to-noise ratio.

### 2.3. Data Collection

Scattered light and fluorescent light produced by the interaction between the light beam and particles or cells are detected by the optical detectors connected to a computer. The most common optical detectors are photomultiplier tubes (PMTs), avalanche photodiodes (APDs), P-doped/Intrinsic/N-doped (PIN) photodiodes [21,26], charge-coupled device (CCD) cameras and CMOS arrays [71,72,73]. PMTs have been widely used in commercial flow cytometers due to the high sensitivity and reliability, especially when dichroic mirrors are used to split the beam for multi-color fluorescence detection. PMTs and other photodiodes can convert incident light to an electrical current and multiply it by as much as $10^{8}$ times. Recently, compact PMTs have been made available on the commercial market, which can be used to replace the old large PMTs and to reduce the overall size of the detection system. Compared to PMTs, APDs are sensitive to temperature, and PIN photodiodes have a much simpler structure. PIN photodiodes can replace PMTs and APDs to reduce the cost when the light signal is strong. PIN photodiodes with lock-in amplification can be utilized for the single cell or particle fluorescence detection [26]. Kettlitz *et al.* avoid expensive PMTs and substitute them with a PIN photodiode, achieving a maximum particle detection frequency of 600 particles/s [74].

Compared to photodiode detectors, CCD camera and CMOS imaging can provide instant images, but the speed of the fluid is limited [51]. Hoera *et al*. utilized a CCD camera for fluorescence imaging of temperature and reaction process in a microfluidic chip reactor [75]. Yang *et al.* conducted an experiment at a flow rate of 10 µL/h to allow CCD cameras to capture the accurate images for real-time cell separation in a microflow cytometer [72]. To further improve the sensitivity of the detector and reduce the cost of the microflow cytometer, Eyer *et al.* [73], added titanium dioxide (TiO${}_{2}$) particles into PDMS to increase the light signal intensity from the interaction between particles and light source, improving the sensitivity of CCD cameras indirectly.

### 2.4. Data Analysis

As shown in Figure 2, light signals collected by optical detectors are further amplified by a current-to-voltage amplifier, and then, the voltage signals are digitized by a data acquisition card. LabView programs can be utilized to record the data for further analysis [20]. Data analysis is usually performed using customized MATLAB codes [20,76].

**Figure 9:**
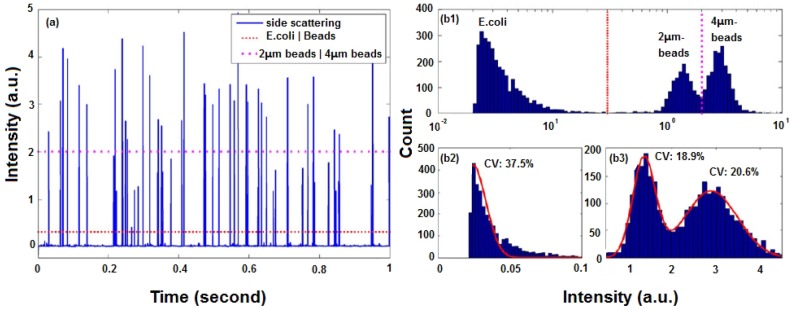
Data analysis results of a mixture of beads and cells flowing in an optofluidic microflow cytometer. (**a**) One second raw data of SSC signal intensity from a test with a mixture of *E. coli* cells and beads of 2 µm and 4 µm in diameter. (**b1**) Statistical histograms of events by total beads and *E. coli* cells with intensity on a logarithmic scale. (**b2**) Statistical histograms of events produced by *E. coli* cells on a linear scale and its Gaussian fitting. (**b3**) Statistical histograms of events by beads with intensity on a linear scale and Gaussian fittings. Reprinted from [20] with permission from Wiley.

When a cell or a particle passes through the interrogation region, a burst of light will generate an electrical pulse in the analysis system. The pulse duration relates to flow rate, the beam waist and the size of cell or particle. FSC intensity is proportional to the size of the cell or particle, and SSC intensity depends on the granularity. Each pulse is characterized by FSC, SSC, single-color or multi-color FL and pulse duration. The average intensity and pulse duration are typically calculated. To remove some background noise or internal PMT noise, a threshold is set. Figure 9a shows the data analysis results of a of mixture beads and cells flowing in an optofluidic microflow cytometer [20]. One dashed threshold is set to distinguish 2- and 4-µm beads; another dotted threshold is set to separate beads and *Escherichia coli* cells.

In flow cytometry, simultaneous detection of multiple parameters is the source of its analytical power. By analyzing and comparing the scattered light signals and fluorescent light signals of a single-cell or single-particle against the total population, it is possible to see the similarities and differences for further counting and identification. Figure 9b shows histograms of events with intensity on a logarithmic scale and a linear scale. Cells and beads of different diameters show various distribution features, which can be fitted by Gaussian curves with different coefficients of variation (CV). For a microflow cytometer with multi-color fluorescence channels, a multi-parameter plot can be used to do further analysis.

## 3. Fabrication and Integration

### 3.1. Materials

Traditionally, silicon and glass substrates are the most common materials used in microflow cytometers. Recently, inorganic materials, like ceramics, and polymers, like PDMS, and even paper [77] have been used to construct microfluidic devices. Silicon and glass technologies, as well as polymer technology have been reviewed in many papers [12,77,78].

Wet or dry etching methods are applied to create microstructures on a silicon or glass substrate, but organic long-chain polymers are attracting more and more attention with the growing interest in fabricating multilayered structures. Polymers are less expensive and convenient for mass production. More importantly, most polymers are optically transparent to visible wavelengths of light and adaptable through chemical modification for bonding to glass or silicon substrates. Polymers can be divided into elastomers and thermoplastics. PDMS, as one of the elastomers, was first used as a substrate in the late 1990s. Since then, PDMS has established itself as the most commonly-used elastomer in microfluidics. Chemical modification of PDMS has allowed the diversification of its application in microfluidics. PDMS structures can be cured on molds at room temperature for microchannels or other microstructures, and PDMS can provide good sealing properties after chemical modification. Zhang *et al.* [79] sealed SU-8 microfluidic channels using PDMS after the N${}_{2}$ plasma treatment. Amino groups generated by N${}_{2}$ plasma on the PDMS surface reacted with the residual epoxy groups on the SU-8 surface. The bond was long-term resistant to water, and the structure could withstand a high degree of stress. Polystyrene (PS), polycarbonate (PC), poly(methyl methacrylate) (PMMA) and cyclic olefin copolymer (COC) are other thermoplastic polymers that are used in microfluidics.

As stated earlier, SU-8 is a commonly-used epoxy-based negative photoresist that can be utilized to form the functional layer (waveguides or lenses) when processed on a substrate [24,67,80]. High aspect ratio structures can be obtained in SU-8 by lithography. Researchers now are seeking methods to integrate PDMS and SU-8 together to take advantage of both materials. Ren *et al.* [80] bonded SU-8 and PDMS using the aminosilane-mediated bonding method in a microfluidic device for neuroscience research. Paper is a promising new material with low cost and easy fabrication process. Furthermore, paper is available everywhere and relatively environmentally-friendly. Liu and Crook [77] fabricated a 3D paper microfluidic devices simply by hand folding. Colorimetric and fluorescence detection of glucose and protein were achieved.

### 3.2. Device Integration

**Figure 10:**
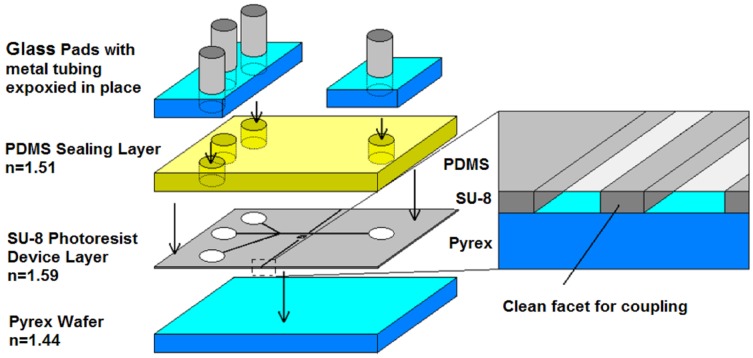
A schematic diagram showing the integration of a multilayered PDMS/SU-8 device. SU-8 is the functional layer; PDMS covers and seals the device; and glass pads allow solid fluidic interconnects. Reproduced from [65] with permission.

**Figure 11:**
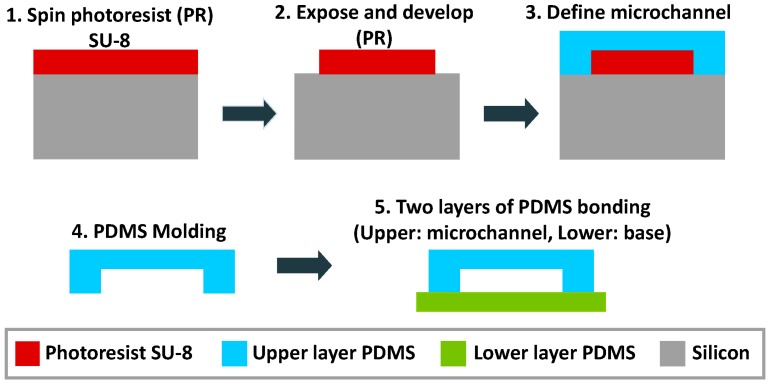
Standard fabrication procedure of a PDMS/SU-8 device. Reprinted from [81] from *Micromachines* published by MDPI.

A multilayered PDMS/SU-8 devices is commonly used in an optofluidic microflow cytometer. Standard techniques, like wet and dry etching on glass or silicon substrates, have been replaced by soft lithography, photolithography and different bonding techniques in recent decades. Figure 10 shows the individual layers for the integration of the device, and the detailed process can be seen in Figure 11. The PDMS layer is fabricated by PDMS molding and functioned as a upper layer to seal the fluidics and to form an upper optical cladding layer. The SU-8 layer is patterned on a silicon or glass substrate by exposure to UV light through photomasks. Due to the high aspect ratio, the waveguides and optical components can be integrated on the SU-8 layer. To assemble the device, the PDMS layer is bonded to the SU-8 layer after a plasma treatment [67,80]. The PDMS cover provides a complete seal, and extra glass pads are bonded on the top surface of PDMS. The sandwiched device is rigid enough and can withstand a high pressure. Custom optical and fluidic components can be integrated on a small chip to achieve specialized and efficient function.

## 4. Performance of Optofluidic Microflow Cytometers

Optofluidic microflow cytometers aim to provide a compact and automated method capable of high-throughput screening, low reagent consumption, high sensitivity and high selectivity for small particles or cells detection. Current researchers are moving forward step by step to achieve those goals and narrowing the performance difference between the conventional benchtop and optofluidic microflow cytometers.

The sensitivity of the optofluidic microflow cytometer is improved by the on-chip lens system. The fluorescence sensitivity of flow cytometers depends on the background noise, effective intensity of light signal and the detection efficiency. SNR is the ability to resolve a pulse from the noise. The minimum SNR ratio for a reliable detection is three, while the SNR value of fluorescent beads measurement was 80–300 from devices by using novel on-chip lens systems and notched designs to reduce the background noise [68].

Table 3 shows the parameters of optofluidic microflow cytometers developed recently and their performance measured by the coefficient of variation (CV). The CV is defined as the ratio of the standard deviation to the mean of the light signal intensity expressed as a percentage. This effectively measures the dispersion of intensity of the detection events [82]. A smaller CV indicates that there is less error introduced by the actual device and that identical samples will have identical detected signals, meaning that the microflow cytometer has a higher ability to differentiate the slight differences of the type of particles or cells in the entire population.

The throughput of optofluidic microflow cytometer varies from 30 particles or cells/s to 2000 cells/s or even to 50,000 cells/s. Different levels of throughputs are achievable depending on the specific application. For *E. coli* or bacteria detection, where the concentrations of targeted cells are very low, a low throughput is used. For rare cell analysis or blood cell counting, high throughputs are required. As shown in Table 3, the CV values for cells are significantly higher than those of blank or fluorescent labeled beads. Since the refractive index contrast between cells and sample fluid (usually water or phosphate-buffered saline) is small, the intensity of scattered light is not as strong as that of beads. Fluorescent labeling is applied to provide an easily detectable parameter to help improve SSC detection by correlating the two parameters; if an FL is detected, then a SSC must be detected, as well. This can indirectly enhance the SNR of the SSC. The CV value of SSC intensity produced by *E. coli* cells was 37.5% [20], while the CV value of FL intensity produced by labeled human embryonic kidney (HEK) cells is 13.4% [38].

An optofluidic microflow cytometer with typical 2D hydrodynamic focusing, on-chip beam shaping and collection was studied by Watts *et al.* [55]. Scattered light signals of 1-, 2- and 5-µm blank beads were collected by on-chip waveguide, obtaining CV values of 16.4%, 11.0% and 12.5%, respectively. It also showed that the performance of optofluidic microflow cytometers is based on the combination of the beam geometry used and bead size. Barat *et al.* [2] demonstrated a 2D hydrodynamic focusing optofluidic microflow cytometer with an on-chip lens system for light guiding and grooves for inserted optical fibers for collection. The flow cytometer successfully differentiates 10–25-µm beads based on fluorescence and scattered light. The best CV value was 4.9% for the side scattered light intensity of 25-µm beads.

The performance of a 2D hydrodynamic focusing optofluidic microflow cytometer with free-space collection was determined to be very comparable to conventional cytometers. Watts *et al.* [54] focused the beam waist to 6 µm, and fluorescent signals from 2.5-µm beads showed a superior CV of 9.03%. Mu *et al.* [24] detected labeled *E. coli* cells, achieving a detection efficiency of 89.7% and 94.5% for fluorescence signals and scattered light signals, respectively. The detection accuracy was 84.3% and 88.8% for fluorescence and scattered light detection, respectively, as compared to the standard haemocytometer method.

Nawaz *et al.* [38] presented a novel microfluidic drifting-based 3D hydrodynamic focusing optofluidic microflow cytometer with free space light collection. The best CV of 2.37% for fluorescent beads was achieved, which is comparable to a commercial benchtop flow cytometer. Frankowski *et al.* [28,29] developed two microfluidic sensors based on optical and impedance analysis both operating simultaneously. They demonstrated a superior CV of 3.2% for 8.12-µm beads. A combination of multiple methods or techniques is a good way to improve the sensitivity of microfluidic devices.

Typically, good 3D hydrodynamic focusing ability in both lateral and vertical directions can achieve a lower CV than 2D focusing. However, beam shaping can also improve the detection efficiency and obtain a lower CV value even with 2D hydrodynamic focusing. A CV of 15.9% for 2-µm beads was achieved by Watts *et al.* [55] using 2D hydrodynamic focusing, while a similar CV of 15.4% for 10-µm beads was obtained in a 3D hydrodynamic focusing made by Testa *et al.* [31]. This is because Watts *et al.* used an on-chip lens system to focus the beam waist to 1.5 µm and formed a superior uniform region of light intensity of the interrogation region [55]. Both devices provide free optical alignment, but the grooved structure in Testa’s device is easier to fabricate.

Besides hydrodynamic focusing techniques, SSAW also provides a good focusing quality. A mixture of 7-µm beads and 10-µm beads was distinguished by an SSAW-based microfluidic cytometer studied by Chen *et al.* [41], with CVs of 19.4% and 10.9%, respectively. Fluorescently-labeled human promyelocytic leukemia cells (HL-60) were successfully detected with a CV of 22.0%.

The typical CV value achievable by the conventional benchtop flow cytometer is about 5%–15%. The performance of a optofluidic microflow cytometer has developed dramatically from 24.73% by Mu *et al.* [24] to 3% by Frankowski *et al.* [29] in the past few years. The performance of an optofluidic microflow cytometer now can be comparable to the conventional benchtop flow cytometer. The improvement of the device’s performance is attributable to the development of microchip devices’ enhanced flow control methods, the on-chip lens system that provides custom and robust beam shaping capabilities, the signal collection method and the rapidly-growing microfabrication techniques.

## 5. Conclusions and Future Perspective

Optofluidic microflow cytometers are attracting more and more research attention, combining multiple fields and disciplines in a microchip. Optofluidic microflow cytometers integrated with optics provide significant enhancements for flow cytometry. Low costs, higher sensitivity, free optical alignment and smooth interaction interfaces are the obvious advantages. Optofluidic microflow cytometers offer significantly lower costs and size reductions, as well as low reagent requirements and portability advantages over a benchtop flow cytometer.

Various attempts have been made to integrate on-chip waveguides or grooves for the guided insertion of optical fibers into the microchip. Besides on-chip lenses or waveguides fabricated by SU-8 or PDMS, novel types of liquid-core/liquid-cladding lenses, liquid-core/liquid-cladding waveguides and hybrid core waveguides have been used in optofluidic devices. The integrated lens system helps shape the beam emitted from the excitation source, providing an optimal geometry and uniform region for interaction with the specimen. By taking advantage of both optics and fluidics, the performance of optofluidic microflow cytometers has advanced to a point where devices are comparable to that of a conventional flow cytometer in the past few years. To the extent of our knowledge, the best CV value achieved for a microflow cytometer was less than 3% [29,38].

Currently, the optofluidic devices have successfully miniaturized the optical and fluidic components of the flow cytometer, while the miniaturization of the whole system is still challenging. System integration needs to have more attention paid to it. External syringe pumps, light collection detectors and other components still somewhat limit the portability of the optofluidic microflow cytometer. The miniaturization of these components needs to join the current stat of the optical and microfluidic control components for continuous development of future POC applications. For POC diagnostic and other field applications, a simple method for mass production should be invented in the future to further reduce the cost. For commercialization, optofluidic microflow cytometers need to provide significant operational advantage over conventional flow cytometers. A fully-portable, low cost, easy to operate and effective optofluidic microflow cytometer can be reasonably expected in both the research field and in the commercial market.

## Figures and Tables

**Table 1:** Terminology.

Terminology	Main Device Used	Description
Microflow cytometer	Microfluidic device	Integrated optics are not necessary
Optofluidic microflow cytometer	Optofluidic device	Integrated optics are necessary

**Table 2:** Techniques related to the performance of an optofluidic microflow cytometer.

Flow Control	Light Guide	Light Collection	Collected Signal
2D hydrodynamic focusing	Free-space/on-chip	Free-space/on-chip	Fluorescence collection (FL)
3D hydrodynamic focusing	Free-space/on-chip	Free-space/on-chip	Side scattered light (SSC)
Other methods	Free-space	Free-space	Forward scattered light (FSC)

**Table 3:** Performance of recently developed optofluidic microflow cytometers.

Flow Control	Beam Shaping	Light Collection	Sample	CV of SSC (%)	CV of FL (%)	Throughput (Cells or Particles/s)	Ref.
2D HF	Yes	Free-space	*E. coli*	37.5	–	∼101	[20]
2D HF	Yes	On-chip	2 µm beads	11	–	∼30	[55]
2D HF	Yes	On-chip	15 µm beads	12	17.1	∼100	[2]
2D HF	No	Free-space	Labeled *E. coli*	36.2	30.7	∼350	[24]
2D HF	No	Free-space	1 µm beads	14.95	24.73	∼83	[24]
2D HF	Yes	Free-space	2.5 µm beads	–	9.0	∼28	[54]
3D HF	No	On-chip	10 µm beads	12	8.3	-	[31]
3D HF (cascade focusing)	No	Free-space	8.12 µm beads	–	3.2	–	[29]
3D HF (microfluidic drifting)	No	Free-space	1.9 µm beads	–	2.4	∼2163	[38]
3D HF (microfluidic drifting)	No	Free-space	HEK 293 cells	–	13.4	–	[38]
3D HF (cascade focusing)	No	Free-space	Beads	–	3.0	–	[28]
3D SSAW	No	Free-space	HL–60 cells	–	22.0	–	[41]
3D SSAW	No	Free-space	7 µm beads	–	19.4	∼772	[41]
3D SSAW	No	Free-space	10 µm beads	–	10.9	∼537	[41]
